# Serum IL8 is not associated with cardiovascular events but with all-cause mortality

**DOI:** 10.1186/s12872-019-1014-6

**Published:** 2019-02-04

**Authors:** Ilais Moreno Velásquez, Ashwini Gajulapuri, Karin Leander, Anita Berglund, Ulf de Faire, Bruna Gigante

**Affiliations:** 1 Unit of Cardiovascular Epidemiology, Institute of Environmental Medicine, Karolinska Institutet, Stockholm, Sweden; 2 Gorgas Memorial Institute for Health Studies, Panama City, Panama; 3 Division of Cardiovascular Medicine, Department of Clinical Sciences, Danderyd University Hospital, Stockholm, Sweden

**Keywords:** Interleukin 8, Cardiovascular disease, All-cause mortality, Cancer mortality, Cardiovascular mortality

## Abstract

**Background:**

The aim of this study is to investigate if IL8 levels were associated with incident cardiovascular (CV) events (CVE) and mortality (all-cause, CV, and cancer) in a cohort of 60 years old men and women from Stockholm (60YO).

**Methods:**

The 60YO comprises 4232 participants; baseline period: 1997–1999. The cohort is matched annually to population registries to record deaths and incident CVE. Serum IL8 was measured in 4011 participants and categorized in quartiles. Cox proportional hazard models were used to estimate the CVE and mortality risk, expressed as hazard ratios (HR) with 95% confidence intervals (CI). Potential confounding was addressed by adjusting for traditional CV risk factors (CVE estimates) and by sex, life style habits, metabolic factors (mortality estimates). Laplace regression was used to calculate the difference in time until a certain percentage of the cohort died according to IL8 levels.

**Results:**

During 16.5 years follow up, 522 incident CVE were recorded and 647 study participants died. IL8 was not associated with CVE risk (IL8 Q4 vs Q1, HR of 0.95; 95% CI 0.75–1.22). Compared to Q1, IL8 Q4 was associated with all-cause mortality (adjusted HR 1.28; 95% CI 1.02–1.63). No association was observed with CV and cancer related mortality in the fully adjusted model. Participants with IL8 above the median died of any cause ≈1.3 years before the 15% of the population had died.

**Conclusion:**

Elevated IL8 levels were not associated with CVE risk and CV mortality, but were associated with an increased risk of all-cause mortality regardless of the underlying cause.

**Electronic supplementary material:**

The online version of this article (10.1186/s12872-019-1014-6) contains supplementary material, which is available to authorized users.

## Background

Chronic inflammation is a hallmark of atherosclerosis and cancer [1, 2]. Among the inflammatory chemokines, Interleukin 8 (IL8) plays a role in atherogenesis and atherosclerotic plaque destabilization. IL8 recruits monocytes into the subendothelial space [3], has mitogenic and chemotactic effects on vascular smooth muscle cells [4] and contributes to plaque vulnerability by promoting an imbalance between metalloproteinases and metallopeptidases [5]. At the same time, in ischemic tissues, IL8 seems to be beneficial by accelerating neovascularization [6] and promoting angiogenesis [7]. In cancer, IL8 is secreted and expressed by various cancer cell types, has a cardinal pro-angiogenic activity on endothelial cells, induces neovascularization and prolonged survival and proliferation of cancer cells [8].

Among atherosclerosis related CVE, the role of IL8 has been investigated as risk indicator for CHD with controversial results. Elevated levels of IL8 were associated with an increased risk of future CHD in the EPIC-Norfolk study [9] while they did not appear as an independent risk factor for CHD in the MONICA/KORA case-cohort study [10] and were associated with a reduced occurrence of MI in the SHEEP case-control study [11].

Circulating IL8 has not been studied in relation to the risk of ischemic stroke in prospective CV studies, but high levels of IL8 in plasma and cerebro-spinal fluid have been observed in the acute phase of stroke [12, 13].

IL8 as a predictor of cancer has been scarcely studied. However, a relation between IL8 and disease progression has been reported in at least ten types of cancers [14].

Little is known with regard to IL8 as a risk marker for cancer and CV mortality. IL8 has been associated with all-cause mortality in elderly women from the PIVUS cohort [15] and in a secondary analysis from the CORONA trial [16], whereas such association was not observed in an elderly community dwellings from the MEMO study [17].

Recent data from the CANTOS study have demonstrated the importance of the resolution of the inflammatory reaction to prevent CVE and cancer [18, 19]. Given the central role of IL8 in initiating the inflammatory response in atherosclerosis, we aimed to investigate the role of IL8 as a predictor for first time atherosclerosis related CVE (myocardial infarction, angina requiring hospitalization, ischemic stroke). Moreover, we also estimated the risk of all-cause, CV and cancer related mortality in relation to circulating IL8. We performed our study in a large cardiovascular cohort, the 60YO, where participants were followed-up until death or first CVE.

## Methods

### Study population

The 60YO was established in 1997–1998 and fully described elsewhere [20, 21]. Briefly, every third man and woman living in Stockholm County born between 1 July 1937 and 30 June 1938 was randomly selected from the population register and invited to participate in a health screening for CVD from August 1997 to March 1999. Of 5460 subjects invited to participate, 4232 (2039 men and 2193 women) agreed to be enrolled (participation rate 78%).

Participants were invited to fill in a questionnaire on life style, diet, past and current medical history and to participate to a health screening where anthropometric measures were recorded. The original version of the questionnaire is in Swedish language; an English translation of the questions used in the present study is shown in Additional file 1. Blood samples were obtained after overnight fasting and stored (− 80 °C) until analyses.

The study and the consent procedure were approved by the Regional Ethical Review Board at Karolinska Institutet and by the Regional Ethical Review Board in Stockholm, Sweden (reference numbers 96–398, 99–306, 03–100, 03–115 and 16/205–31/2). Participants gave their informed consent orally to be enrolled in the study, since written consent forms were not in current use when the study was initiated. All clinical investigations were conducted according to the principles expressed in the Declaration of Helsinki.

### Outcome ascertainment

The 60YO cohort is annually matched with the Swedish National Patient Register and the Cause of Death Register where diagnoses are registered using the International Classification of Diseases (ICD) 10th Revision codes. In this study, only main diagnoses registered until December 31st 2014 were extracted to ascertain the outcome. Follow-up was 99.8%; the linkage to mandatory high-quality population-based registers minimizes the possibility of differential loss to follow-up.

Participants without IL8 measurement (*n* = 103) and with incomplete questionnaires (*n* = 118) were excluded, leaving 4011 participants (Additional file 2: Figure S1).

Atherosclerosis related CVE were recorded using the diagnosis codes for MI (I21), angina requiring hospitalization (I20 and I25), and ischemic stroke (I63). To analyze the association of IL8 with the risk of first CVE, we excluded participants with prior CVE (*n* = 362), i.e. those who had reported a former CVE in the questionnaire and/or those where a CVE diagnosis was recorded in the register before the enrollment date. Furthermore, we excluded participants with the following diagnoses codes: I46-sudden cardiac death since the underlying cause was unknown (*n* = 4), I64.9-stroke not specified as hemorrhage or infarction (*n* = 6), I65.2- carotid artery stenosis since it was unclear if they had an ischemic stroke (*n* = 8), I25.2-old MI (*n* = 1) and I25.9-chronic ischemic heart disease (*n* = 4) since we could not verify the time at which the event occurred. After exclusion, 522 incident CVE were analyzed (Additional file 2: Figure S1, left side of the flowchart). In this group, CVE was the cause of death in 67 cases.

Six-hundred-forty-seven participants died until December 31st 2014. Death was defined as CV related if the ICD code that defined the underlying cause of death belonged to the I00–99 diagnosis code group. After the exclusion of prevalent CVE, 136 cases were analyzed. Death was considered as cancer related if the ICD code belonged to the C00-C99 group. A prior cancer diagnosis was reported in the questionnaire by 115 study participants, which were excluded, leaving 266 cases in the analysis (Additional file 2: Figure S1, right side of the flowchart).

### IL8 measurements

Serum samples were retrieved from frozen storage, thawed, and IL8 levels were measured using electrochemiluminescence immunoassay plates produced by Meso-Scale Discovery’s Multi-Array® (MSD). Concentrations were derived from the standard curve and expressed as picograms per milliliter (pg/mL). Intra-assay variability (5.8%) was calculated as the average of the coefficients of variation of duplicate samples (*n* = 544) run in the same assay. Inter-assay variability (2.8%) was calculated as the average of coefficient of variation of duplicate pooled samples run in 15 consecutive plates. According to the manufacturer, mean intra-assay coefficient of variation should not exceed 15% and mean inter-assay 18%. Samples were analyzed in a random order and blinded with regards to case-non-case status in order to avoid observer expectation bias.

### Clinical and anthropometric variables: Definition of potential confounding factors

Systolic- and diastolic blood pressure (BP) were measured as previously reported [21]. Hypertension was defined as BP ≥140/90 and/or antihypertensive drug therapy and/or self-reported. Body Mass Index (BMI) was calculated using the formula weight (kg)/ height (cm2). A waist/hip ratio ≥ 0.90 in men and ≥ 0.85 in women was used to define central obesity [21].

Physical activity at work was defined as low (sitting almost all day), medium (sitting half day), medium high (sitting less than half day), high (almost never sitting). Physical activity during leisure time was defined as low (< 2 h/week), medium (about 2 h/week), medium high (30 min 1–2 times/week), high (30 min > 2 times/week).

Smokers were categorized as current smokers or non-current smokers. Daily intake of alcohol in grams was estimated from the average number of cans/bottles drunk daily [22].

Glucose was measured with an enzymatic colorimetric test (Bayer Diagnostics, Tarrytown, NY). Diabetes was defined at baseline as fasting glucose ≥7.0 mmol/L and/or antidiabetic medication intake and/or self-reported. Cholesterol and triglycerides were analyzed using enzymatic methods (Bayer Diagnostics, Tarrytown, NY). Hypercholesterolemia was defined as fasting total cholesterol > 5.0 and/or treatment for hypercholesterolemia and/or self-reported.

### Statistical analysis

Baseline characteristics of the study population are reported according to IL8 quartiles (Q1 ≤ 8.7, Q2 > 8.7 ≤ 11.9, Q3 > 11.9 ≤ 17.3, Q4 > 17.3). Continuous variables are presented as median and interquartile range (IQR).

We investigated the association of IL8 as a continuous variable and categorized in quartiles with the risk of first CVE and with the risk of all-cause, CV- and cancer- related mortality (IL8Q1 as the reference group). Cox proportional hazards model was used to calculate hazard ratios (HR) with 95% confidence intervals (CI).

In the association of IL8 with the risk of first atherosclerosis related CVE, estimates were adjusted by sex, hypertension, diabetes, hypercholesterolemia, smoking and central obesity. In the association of IL8 with mortality risk, the influence of potential confounding was addressed by adjusting for sex and life style factors (smoking, alcohol consumption, physical activity at work and during leisure time) in model 1a and by the addition of the common cardiometabolic risk factors (waist/hip ratio, systolic and diastolic blood pressure, glucose and cholesterol levels) in model 1b. Schoenfeld’s test confirmed proportionality of hazards.

Laplace quantile regression was used to calculate the difference in time (in years) until a certain proportion of the participants had died during follow-up according to IL8 levels. We used 5th, 10th, 15th percentile of the study population, given that almost 15% of the individuals in our cohort had died over the entire follow-up. In this analysis, IL8 levels were dichotomized at the median. Risk estimates were adjusted according to the model 1b described above.

All calculations were performed using Stata v.14.

## Results

Table 1 summarizes the distribution of baseline characteristics observed per IL8 quartile. With increasing IL8 quartiles study participants were mostly men and current smokers, had more frequently a history of diabetes, central obesity and hypercholesterolemia. Similar values in cholesterol and glucose levels were observed across IL8 quartiles, as well as in reported physical activity at work and during leisure time.

**Table 1:** Baseline characteristics of study population according to IL8 quartiles

	IL8 Q1 (IL8 ≤ 8.7 pg/ml)	IL8 Q2 (IL8 > 8.7 ≤ 11.9 pg/ml)	IL8 Q3 (IL8 > 11.9 ≤ 17.3 pg/ml)	IL8 Q4 (IL8 > 17.3 pg/ml)
Male/Female	438/563	426/576	482/522	595/409
SBP (mmHg)	134 (121–150)	137 (122–152)	136 (123–152)	137 (123–152)
DBP (mmHg)	83 (76–90)	83 (76–91)	83 (76–91)	84 (77–91)
Anthropometric measures				
BMI(kg/cm2)	26 (24–29)	26 (24–29)	26 (24–29)	26 (24–29)
Waist/hip ratio	0.8 (0.8–0.9)	0.8 (0.8–0.9)	0.9 (0.8–0.9)	0.9 (0.8–1.0)
Life style factors (%)				
Physical activity at work				
Low	312 (24)	321 (25)	338 (26)	312 (24)
Medium	212 (24)	225 (25)	213 (24)	225 (25)
Medium high	227 (24)	236 (25)	230 (25)	233 (25)
High	210 (24)	205 (24)	220 (26)	209 (25)
Physical activity during leisure time				
Low	100 (22)	123 (27)	111 (24)	121 (27)
Medium	594 (26)	569 (25)	570 (25)	565 (25)
Medium high	220 (24)	225 (25)	239 (26)	220 (24)
High	62 (21)	75 (25)	85 (28)	73 (25)
Alcohol consumption (g/day)	7.8 (2.2–15.2)	8.0 (2.2–17.6)	8.1 (2.7–16.8)	10.2 (4.2–21.1)
Cardiovascular risk factors (%)				
Smoking	530 (22)	594 (25)	608 (25)	647 (27)
Diabetes	19 (14)	36 (26)	41 (30)	43 (31)
Hypertension	193 (24)	181 (23)	218 (27)	197 (25)
Hypercholesterolemia	39 (18)	46 (22)	66 (31)	61 (29)
Central obesity	487 (23)	505 (24)	539 (25)	585 (27)
Biochemical markers (mmol/L)				
Cholesterol	6.0 (5.3–6.6)	6.0 (5.3–6.7)	6.0 (5.3–6.7)	6.0 (5.3–6.7)
LDL-cholesterol	3.9 (3.3–4.5)	3.8 (3.3–4.5)	3.9 (3.3–4.5)	3.8 (3.2–4.4)
Glucose	5.2 (4.8–5.6)	5.2 (4.8–5.6)	5.2 (4.8–5.7)	5.2 (4.9–5.7)

Continuous variables are presented as median and interquartile range. Abbreviations: SBP: systolic blood pressure; DBP: diastolic blood pressure; BMI body mass index, LDL low density lipoproteins, CVE cardiovascular events. Missing values: SBP and DBP, *n* = 3; waist/hip ratio, *n* = 2; physical activity at work, *n* = 83; physical activity leisure time, *n* = 59; smoking, *n* = 57; alcohol consumption, *n* = 1; LDL, *n* = 47

### IL8 and risk of first CVE

During a follow-up period of 16.5 (15.9–16.8) years, 522 incident CVE were recorded: 177 MI, 181 angina requiring hospitalization and 164 ischemic stroke. Exposure to increasing IL8 levels indicated no association between IL8 and first CVE (adjusted HR of 0.99 (0.90–1.00). After categorization in quartiles, no differences in CVE risk were observed across IL8 quartiles (adjusted HR of 0.96; 95% CI 0.75–1.22, IL8 Q4 vs IL8Q1). Similarly, IL8 levels were not associated with the risk of MI and angina requiring hospitalization or ischemic stroke after adjustment for the traditional CV risk factors (Table 2 and Additional file 3: Figure S2, Additional file 4: Figure S3 and Additional file 5: Figure S4).

**Table 2:** Association between serum IL8 levels with the risk of first CVE and CVD related death expressed as hazard ratios (HR), 95% confidence intervals (CI)

	IL8Q1	IL8Q2	p	IL8Q3	p	IL8Q4	p
CVE (*n* = 522)	776/125	782/127		776/128		765/142	
Crude (*n* = 3626)	1	1.01 (0.78–1.29)	0.94	1.03 (0.80–1.31)	0.81	1.15 (0.90–1.46)	0.24
Model 1 (*n* = 3583)	1	0.98 (0.76–1.25)	0.87	0.94 (0.73–1.20)	0.63	0.96 (0.75–1.22)	0.74
MI and angina requiring hospitalization (n = 358)	778/91	783/82		779/94		764/91	
Crude (*n* = 3462)	1	0.90 (0.67–1.21)	0.51	1.03 (0.78–1.38)	0.80	1.03 (0.77–1.38)	0.82
Model 1 (*n* = 3417)	1	0.88 (0.65–1.20)	0.44	0.93 (0.70–1.24)	0.63	0.83 (0.62–1.24)	0.23
Ischemic stroke (*n* = 164)	778/34	783/45		779/33		764/52	
Crude (*n* = 3462)	1	1.30 (0.83–2.03)	0.24	0.97 (0.60–1.57)	0.91	1.55 (1.01–2.40)	0.04
Model 1 (*n* = 3226)	1	1.24 (0.79–1.94)	0.34	0.91 (0.56–1.48)	0.73	1.37 (0.88–2.12)	0.15

Number of study participants in each IL8 quartile refers to the crude model. Ischemic stroke cases (*n* = 164) were excluded from the analysis of the association of IL8 with the risk of MI and angina requiring hospitalization. MI and angina requiring hospitalization (*n* = 358) were excluded from the analysis of the association of IL8 with the risk of ischemic stroke. Missing values in the confounders are specified in Table 1

Model 1: adjusted by sex, smoking, diabetes, hypercholesterolemia, hypertension, diabetes and central obesity

Exposure to the highest IL8 quartile was associated with an increased risk of fatal CVE (*n* = 67) (HR of 2.55; 95% CI 1.22–5.31), however the estimate did not attain statistical significance after adjustment for the CV risk factors (IL8 Q4 vs Q1, HR of 1.87 and 95% CI 0.89–3.94).

### IL8 and risk of all-cause, cardiovascular and cancer related mortality

We did not observe an increased risk of all-cause mortality (adjusted HR 1.00; 95% CI 0.99–1.01), CV mortality (adjusted HR 1.00; 95% CI 0.98–1.01) and cancer related mortality (adjusted HR 0.99; 95% CI 0.98–1.01) per unit increase in serum IL8.

However, as shown in Table 3 and Additional file 6: Figure S5, Additional file 7: Figure S6 and Additional file 8: Figure S7, compared to the reference category (IL8Q1), high IL8 levels were associated with an increased risk of all-cause mortality (adjusted HR 1.28; 95% CI 1.02–1.63). In the crude models, high levels were also associated with an increased risk of CV and cancer related mortality. However, estimates did not attain statistical significance in the fully adjusted model.

**Table 3:** Association between serum IL8 levels and risk of all cause, CVD and cancer related mortality expressed as hazard ratios (HR), 95% confidence intervals (CI)

	IL8Q1	IL8Q2	*p*	IL8Q3	*p*	IL8Q4	*p*
All-cause mortality (*n* = 647)	869/127	849/153		843/178		803/189	
Crude (*n* = 4011)	1	1.21 (0.95–1.53)	0.11	1.40 (1.11–1.76)	0.004	1.54 (1.23–1.93)	< 0.0001
Model 1a (*n* = 3845)	1	1.13 (0.89–1.45)	0.30	1.34 (1.06–1.70)	0.01	1.35 (1.07–1.71)	0.01
Model 1b (*n* = 3837)	1	1.09 (0.85–1.39)	0.38	1.27 (1.01–1.62)	0.04	1.28 (1.02–1.63)	0.03
CVD related mortality (*n* = 136)	885/24	881/38		878/32		868/42	
Crude (*n* = 3648)	1	1.58 (0.95–2.64)	0.07	1.35 (0.79–2.29)	0.26	1.80 (1.01–2.97)	0.02
Model 1a (*n* = 3509)	1	1.48 (0.80–2.32)	0.15	1.35 (0.77–2.36)	0.28	1.48 (0.87–2.53)	0.14
Model 1b (*n* = 3504)	1	1.37 (0.79–2.38)	0.25	1.24 (0.71–2.17)	0.44	1.35 (0.79–2.32)	0.26
Cancer related mortality (*n* = 266)	906/55	903/68		934/64		887/79	
Crude (*n* = 3896)	1	1.23 (0.89–1.78)	0.18	1.15 (0.80–1.65)	0.44	1.46 (1.04–2.07)	0.03
Model 1a (*n* = 3732)	1	1.18 (0.82–1.71)	0.36	1.09 (0.75–1.59)	0.62	1.27 (0.89–1.82)	0.18
Model 1b (*n* = 3724)	1	1.15 (0.79–1.66)	0.45	1.06 (0.73–1.53)	0.75	1.23 (0.86–1.77)	0.24

Numbers of study participants is reported for the crude model. Missing values in the confounders are specified in Table 1. Model 1a: adjusted by sex, smoking, alcohol consumption, physical activity at work and during leisure time; Model 1b: model 1a + systolic and diastolic blood pressure, central obesity, cholesterol and glucose levels

We estimated the difference in time to death according to IL8 levels (Table 4). Study participants with IL8 levels higher than the median value (Q2) died, regardless of the underlying cause, about 1.4 and 1.3 year before the 10th and 15th percentile of the population had died. A similar trend, although not statistically significant, was observed when CV and cancer related mortality were analyzed separately.

**Table 4:** Laplace regression model showing the difference in time (years) until the 5th, 10th and 15th of study participants exposed to serum IL8 levels above the median had died (all cause, CV and cancer mortality) as compared to those with serum IL8 levels below the median

Percentile	All-cause mortality		Cardiovascular mortality		Cancer mortality	
	Years (95% CI)	p	Years (95% CI)	p	Years (95% CI)	p
5th	−0.78 (−4.15–2.58)	0.64	0.09 (−2.73–2.55)	0.94	− 0.51 (−1.50–0.46)	0.30
10th	−1.45 (− 2.96–0.05)	0.05	− 0.55 (− 2.58–1.47)	0.59	−0.52 (− 1.09–0.04)	0.07
15th	−1.28 (− 2.43- -0.12)	0.03	− 0.66 (− 2.50–1.17)	0.47	−0.14 (− 0.43–0.15)	0.33

Data in the table represent the results of the fully (model 1b described in Table 3) adjusted regression model

## Discussion

The main finding of the present study is that elevated levels of IL8 were not associated with the risk of future CVE. On the other hand, IL8 levels were associated with an increased risk of all-cause mortality independently from life-style and cardiometabolic risk factors.

Previous studies performed in European cohorts have challenged the hypothesis that IL8 might represent a biomarker for CVD risk. High levels of IL8 were associated with the risk of future CHD, defined as fatal and non-fatal CHD in individuals aged 45–79 years after 6 years of follow-up in a UK population [9] whereas IL8 were not associated with incident CHD, defined as incident fatal, non-fatal MI and sudden cardiac death, in a German population below the age of 75 years after 11 years of follow-up [10]. Conversely, in a large MI case-control study we have observed a reduced occurrence of non-fatal MI in individuals exposed to elevated levels of IL8, reverse causality being one of the possible explanations of these results [12]. Unlike previous studies, our CVE definition included angina pectoris requiring hospitalization and ischemic stroke, conditions that accounts for approximately 30% each of the incident events. While IL8 levels have been reported to be higher in unstable angina pectoris as compared to stable angina pectoris and controls in small clinical studies [23, 24], measurements were performed at the disease onset, and thus, it is likely that IL8 levels mirrored an acute inflammatory state. We did not observe an association of IL8 with risk of ischemic stroke or MI/angina when analyzed separately, therefore the use of composite outcome might not be the sole explanation for the heterogeneity in epidemiological findings reported so far. One relevant possibility in the different findings for the association of IL8 with CV risk across studies might be the inclusion/exclusion of CV deaths. In the present study, we did not observe an association of IL8 with CV related mortality, but the relation between IL8 and fatal atherosclerosis related CVE deserve further exploration. All together, these results suggest that the association of IL8 with risk of CVE might depend upon the baseline CV risk of the population investigated, the length of the follow-up period and the outcome definition that may vary across studies, a phenomenon observed for other inflammatory biomarkers that participates in the early phase of atherogenesis, such as adiponectin [25]. Furthermore, genetic variants associated with IL8 in serum do not associate with the risk of MI [26], suggesting that IL8 can be considered as risk indicator for CVE in certain populations, but not a risk factor for CVE.

Our results on the association of IL8 with mortality regardless of the underlying cause confirm and extend previous observations. IL8 have been shown to predict all-cause mortality in a cohort of 70 year old Swedish women [15] and in a secondary analysis from the CORONA trial [16]. In contrast, elevated levels of IL8 were not a predictor of all-cause mortality in the MEMO study [17] even if higher levels were observed in participants who died during the follow-up as compared to survivors. As opposed to the MEMO study, we did not consider interleukin 6 (IL-6) a confounding factor, since IL8 indirectly regulates IL6 inflammatory effects by prompting the shedding of the soluble IL6 receptor [27] thus participating to the inflammatory reaction stimulated by IL8.

Ageing has been positively correlated with IL8 [10] and reported to increase the visceral adipose tissue prompting secretion of IL8, which is higher in visceral adipose compared to subcutaneous adipose tissue [28]. In fact, previous epidemiological studies in the elderly have underscored the premise that risk estimation in elderly people may warrant different approaches than in young and middle aged subjects [29]. In this respect, high IL8 may reflect a physiological state of biological ageing. Of note, our findings also indicate that high IL8 is associated with an increased risk of early death regardless of the underlying cause. Taken together, these observations suggest that IL8 levels may mirror a chronic inflammatory state already present years before the cancer or CV disease diagnosis that predisposes the individual to an increased risk of death.

This study has strength and limitations. To our knowledge, this is the largest study reported so far on the association of IL8 and CV outcomes. The strength includes the high response rate of those invited to participate and the almost complete long term follow-up of subjects through linkage to high-quality national registries. Our findings are not affected by the potential effect of age on IL8 since participants had the same age at study start. Several limitations deserve mentioning. This study is observational, and hence, residual, unmeasured (e.g., C reactive protein) or unknown potential confounding factors cannot be ruled out. The 60Y0 was designed to identify novel risk factors and biomarkers for cardiovascular diseases. We cannot exclude that a larger cohort would have been necessary to identify the association of IL8 with cancer related mortality. Furthermore, this study may be subjected to regression dilution bias since the potential individual variability of IL8 levels during the follow-up period is unknown. Had misclassification occurred, it would probably be non-differential and thus would bring associations towards the null.

## Conclusions

In conclusion, high IL8 levels may not be considered a risk marker for the risk of future CVE, but they possibly reflect subclinical disease or biological ageing and are therefore associated with an increased risk of death regardless of the underlying cause. The janus properties of IL8, pro-inflammatory and anti-ischemic, observed in experimental studies [30], might partly contribute to explain the discordant association of IL8 with the risk of atherosclerosis related CVEs reported in observational studies. Further larger observational studies aimed to determine the role of this cytokine in the initial stage of atherogenesis will be of interest.

## Additional files

Additional file 1: English translation of the questions used in the present study. All study participants were asked to fill in a questionnaire on life style habits and health status. The original version of the questionnaire is in Swedish. Here it follows the translation of the questions of the questionnaire used in the present study reported as summative data in Table 1. (DOCX 18 kb)

Additional file 2: **Figure S1.** Flowchart of the Cohort of 60 years old men and women from Stockholm. Flowchart summarizing the inclusion and exclusion criteria utilized in the present study. Study participants who did not complete the questionnaire and with missing IL8 serum levels were excluded. The left side of the figure shows the exclusions applied for the analysis of the risk of first atherosclerosis related CVE. The right side of the figure shows the exclusions applied to estimate the risk of CV and cancer related mortality. (DOCX 30 kb)

Additional file 3: **Figure S2;** Graphical representation of the results of the association of serum IL8 with the risk of cardiovascular events. Risk estimate are reported in Table 2. IL8quartile = 0 corresponds to IL8Q1; IL8quartile = 1 corresponds to IL8Q2; IL8quartile = 2 corresponds to IL8Q3; IL8quartile = 3 corresponds to IL8Q4. Panel A: crude model. Panel B: adjusted by sex, smoking, hypertension, diabetes mellitus, hypercholesterolemia, central obesity. Missing values in the confounders are specified in Table 1. (DOCX 34 kb)

Additional file 4: **Figure S3.** Graphical representation of the results of the association of serum IL8 with the risk of myocardial infarction and angina requiring hospitalization. Risk estimates are reported in Table 2. IL8quartile = 0 corresponds to IL8Q1; IL8quartile = 1 corresponds to IL8Q2; IL8quartile = 2 corresponds to IL8Q3; IL8quartile = 3 corresponds to IL8Q4. Panel A: crude model. Panel B: adjusted by sex, smoking, hypertension, diabetes mellitus, hypercholesterolemia, central obesity. Missing values in the confounders are specified in Table 1. (DOCX 34 kb)

Additional file 5: **Figure S4.** Graphical representation of the results of the association of serum IL8 with the risk of ischemic stroke. Risk estimates are reported in Table 2. IL8quartile = 0 corresponds to IL8Q1; IL8quartile = 1 corresponds to IL8Q2; IL8quartile = 2 corresponds to IL8Q3; IL8quartile = 3 corresponds to IL8Q4. Panel A: crude model. Panel B: adjusted by sex, smoking, hypertension, diabetes mellitus, hypercholesterolemia, central obesity. Missing values in the confounders are specified in Table 1. (DOCX 32 kb)

Additional file 6: **Figure S5.** Graphical representation of the results of the association of serum IL8 with the risk of all cause mortality. Risk estimate are reported in Table 3. IL8quartile = 0 corresponds to IL8Q1; IL8quartile = 1 corresponds to IL8Q2; IL8quartile = 2 corresponds to IL8Q3; IL8quartile = 3 corresponds to IL8Q4. Panel A: crude model. Panel B: model 1a, adjusted by sex, smoking, alcohol consumption, physical activity at work and during leisure time; Panel C: model 1b: model 1a + systolic and diastolic blood pressure, central obesity, cholesterol and glucose levels. Missing values in the confounders are specified in Table 1. (DOCX 42 kb)

Additional file 7: **Figure S6.** Graphical representation of the results of the association of serum IL8 with the risk of cardiovascular related mortality. Risk estimate are reported in Table 3. IL8quartile = 0 corresponds to IL8Q1; IL8quartile = 1 corresponds to IL8Q2; IL8quartile = 2 corresponds to IL8Q3; IL8quartile = 3 corresponds to IL8Q4. Panel A: crude model. Panel B: model 1a, adjusted by sex, smoking, alcohol consumption, physical activity at work and during leisure time; Panel C: model 1b: model 1a + systolic and diastolic blood pressure, central obesity, cholesterol and glucose levels. Missing values in the confounders are specified in Table 1. (DOCX 38 kb)

Additional file 8: **Figure S7.** Graphical representation of the results of the association of serum IL8 with the risk of cancer related mortality. Risk estimate are reported in Table 3. IL8quartile = 0 corresponds to IL8Q1; IL8quartile = 1 corresponds to IL8Q2; IL8quartile = 2 corresponds to IL8Q3; IL8quartile = 3 corresponds to IL8Q4. Panel A: crude model. Panel B: model 1a, adjusted by sex, smoking, alcohol consumption, physical activity at work and during leisure time; Panel C: model 1b: model 1a + systolic and diastolic blood pressure, central obesity, cholesterol and glucose levels. Missing values in the confounders are specified in Table 1. (DOCX 45 kb)

## Abbreviations

60YO: Cohort of 60 years old men and women from Stockholm
BMI: Body mass index
CANTOS: Canakinumab Anti-inflammatory Thrombosis Outcomes Study
CHD: Coronary heart disease
CI: Confidence interval
CORONA: Controlled Rosuvastatin Multinational Trial in Heart Failure
CV: Cardiovascular
CVD: Cardiovascular disease
CVE: Cardiovascular events
EPIC: European Prospective Investigation into Cancer and Nutrition
HR: Hazard ratio
IL8: Interleukin 8
IQR: Interquartile range
MEMO: Memory and Morbidity in Augsburg Elderly
MI: Myocardial infarction
MONICA/KORA: Multinational Monitoring of trends and determinants in cardiovascular disease/ Cooperative Health Research in the Region Augsburg
PIVUS: The Prospective Investigation of the Vasculature in Uppsala Seniors
SHEEP: Stockholm Heart Epidemiology Program
