# Supplementary material for: Serum IL8 is not associated with cardiovascular events but with all-cause mortality
Source: BMC Cardiovasc Disord. 2019 Feb 4;19:34. doi: 10.1186/s12872-019-1014-6 (PMC6360748; doi:10.1186/s12872-019-1014-6)
Supplement: Supplementary file 1 — English translation of the questions used in the present study. All study participants were asked to fill in a questionnaire on life style habits and health status. The original version of the questionnaire is in Swedish. Here it follows the translation of the questions of the questionnaire used in the present study reported as summative data in Table 1. (DOCX 18 kb) [file 12872_2019_1014_MOESM1_ESM.docx]

**SUPPLEMENTARY FILE 1.**

**PHYSICAL ACTIVITY**

1. **PHYSICAL ACTIVITY AT WORK.**

How much time do you spend sitting or standing when your work?

1. I sit almost all day (level of activity defined as low in Table 1)
2. I sit about half day (level of activity defined as medium in Table 1)
3. I sit less than half day (level of activity defined as medium-high in Table 1)
4. I never sit (level of activity defined as high in Table 1)
5. **PHYSICAL ACTIVITY DURING LEISURE TIME**

How much have you been active and how much have you been training during leisure time during the last year?

1. You spend most of your free time watching television, film or reading. You walk, bicycle or are active less than 2 hours/week (level of activity defined as low in Table 1);
2. You walk, bicycle or are active at least 2 hours/week without sweating in most cases. Here is included to walk or to bicycle to and from work, walks on holiday, gardening, fishing, table tennis and bowling (level of activity defined as medium in Table 1);
3. You train regularly 1-2 times/week at least 30 minutes/training session with jogging, swimming, tennis, or any activity that make you sweat (level of activity defined as medium-high in Table 1)
4. You train regularly 3 times/week at least 30 minutes/training session with jogging, swimming, tennis, or any activity that make you sweat (level of activity defined as high in Table 1)

**SMOKING**

1. Do you smoke? Yes/No
2. Have you smoked? Yes/no

**ALCOHOL CONSUMPTION**

1. How much light beer do you usually drink?
2. 1 can/bottle per day or more
3. 4-6 cans/bottles per week
4. 2-3 cans/bottles per week
5. 1 can/ bottle per week or less
6. I never drink
7. How much beer do you usually drink?
8. 1 can/bottle per day or more
9. 4-6 cans/bottles per week
10. 2-3 cans/bottles per week
11. 1 can/ bottle per week or less
12. I never drink
13. How much strong beer do you usually drink?
14. 1 can/bottle per day or more
15. 4-6 cans/bottles per week
16. 2-3 cans/bottles per week
17. 1 can/ bottle per week or less
18. I never drink
19. How much wine do you usually drink?
20. 4 bottles per week or more
21. 2-3 bottles per week
22. ½ -1 bottle per week
23. Up to ½ bottle per week or less
24. Few glass of wine per month
25. I never drink
26. How much strong spirit do you usually drink?
27. 1 bottle (75 cl) per week or more
28. 35-75 cl per week
29. 15-35 cl per week
30. 1-15 cl per week
31. I never drink

**MEDICAL HISTORY**

1. Have you previously been in good health?

Check the box if you have had any of the following health related problems. Indicate also in which year you had the disease.

1. Myocardial infarction
2. Angina (chest pain)
3. Heart failure
4. Pain in the legs
5. Diabetes

If you have diabetes do you have any of the following complications?

- Reduced eyesight
- Chronic wound
- Kidney problems
- Amputation

1. High blood pressure
2. High levels of cholesterol
3. Stroke
4. Blood clots in arm/legs/lungs
5. Other diseases?

If you have answered “YES” please specify which disease have you had.
