# Supplementary material for: Serum IL8 is not associated with cardiovascular events but with all-cause mortality
Source: BMC Cardiovasc Disord. 2019 Feb 4;19:34. doi: 10.1186/s12872-019-1014-6 (PMC6360748; doi:10.1186/s12872-019-1014-6)

On Line Supplementary Material.

# Serum IL8 is not associated with cardiovascular events but with all-cause mortality.

Ilais Moreno Velásquez^1,2^, Ashwini Gajulapuri ^1^, Karin Leander^1^, Anita Berglund^1^, Ulf de Faire^1^, Bruna Gigante^1, 3^.

*^1^Unit of Cardiovascular Epidemiology, Institute of Environmental Medicine, Karolinska Institutet, Stockholm, Sweden,^2^* *Gorgas Memorial Institute for Health Studies, Panama,^3^Division of Cardiovascular Medicine, Department of Clinical Sciences, Danderyd University Hospital, Stockholm, Sweden.*

Index.

Supplemental File I

Supplemental Figure I

Supplemental Figure II

Supplemental Figure III

Supplemental Figure IV

Supplemental Figure V

Supplemental Figure VI

Supplemental Figure VII

# Figure S1.


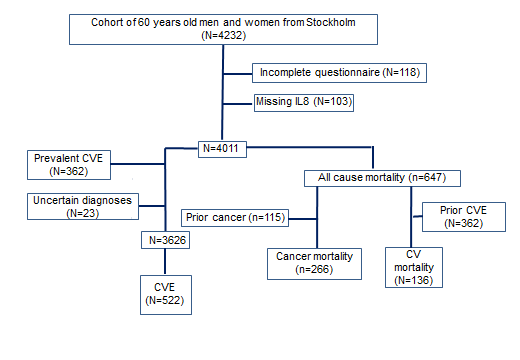

Supplement: Supplementary file 2 — Figure S1. Flowchart of the Cohort of 60 years old men and women from Stockholm. Flowchart summarizing the inclusion and exclusion criteria utilized in the present study. Study participants who did not complete the questionnaire and with missing IL8 serum levels were excluded. The left side of the figure shows the exclusions applied for the analysis of the risk of first atherosclerosis related CVE. The right side of the figure shows the exclusions applied to estimate the risk of CV and cancer related mortality. (DOCX 30 kb) [file 12872_2019_1014_MOESM2_ESM.docx]
