# Supplementary material for: Serum IL8 is not associated with cardiovascular events but with all-cause mortality
Source: BMC Cardiovasc Disord. 2019 Feb 4;19:34. doi: 10.1186/s12872-019-1014-6 (PMC6360748; doi:10.1186/s12872-019-1014-6)
Supplement: Supplementary file 4 — Figure S3. Graphical representation of the results of the association of serum IL8 with the risk of myocardial infarction and angina requiring hospitalization. Risk estimates are reported in Table 2. IL8quartile = 0 corresponds to IL8Q1; IL8quartile = 1 corresponds to IL8Q2; IL8quartile = 2 corresponds to IL8Q3; IL8quartile = 3 corresponds to IL8Q4. Panel A: crude model. Panel B: adjusted by sex, smoking, hypertension, diabetes mellitus, hypercholesterolemia, central obesity. Missing values in the confounders are specified in Table 1. (DOCX 34 kb) [file 12872_2019_1014_MOESM4_ESM.docx]

**Figure S3.** Graphical representation of the results of the association of serum IL8 with the risk of myocardial infarction and angina requiring hospitalization. Risk estimates are reported in Table 2.

A

B
