# Supplementary material for: Serum IL8 is not associated with cardiovascular events but with all-cause mortality
Source: BMC Cardiovasc Disord. 2019 Feb 4;19:34. doi: 10.1186/s12872-019-1014-6 (PMC6360748; doi:10.1186/s12872-019-1014-6)
Supplement: Supplementary file 5 — Figure S4. Graphical representation of the results of the association of serum IL8 with the risk of ischemic stroke. Risk estimates are reported in Table 2. IL8quartile = 0 corresponds to IL8Q1; IL8quartile = 1 corresponds to IL8Q2; IL8quartile = 2 corresponds to IL8Q3; IL8quartile = 3 corresponds to IL8Q4. Panel A: crude model. Panel B: adjusted by sex, smoking, hypertension, diabetes mellitus, hypercholesterolemia, central obesity. Missing values in the confounders are specified in Table 1. (DOCX 32 kb) [file 12872_2019_1014_MOESM5_ESM.docx]

**Figure S4.** Graphical representation of the results of the association of serum IL8 with the risk of ischemic stroke. Risk estimates are reported in Table 2.

A

B
