# Supplementary material for: Serum IL8 is not associated with cardiovascular events but with all-cause mortality
Source: BMC Cardiovasc Disord. 2019 Feb 4;19:34. doi: 10.1186/s12872-019-1014-6 (PMC6360748; doi:10.1186/s12872-019-1014-6)
Supplement: Supplementary file 7 — Figure S6. Graphical representation of the results of the association of serum IL8 with the risk of cardiovascular related mortality. Risk estimate are reported in Table 3. IL8quartile = 0 corresponds to IL8Q1; IL8quartile = 1 corresponds to IL8Q2; IL8quartile = 2 corresponds to IL8Q3; IL8quartile = 3 corresponds to IL8Q4. Panel A: crude model. Panel B: model 1a, adjusted by sex, smoking, alcohol consumption, physical activity at work and during leisure time; Panel C: model 1b: model 1a + systolic and diastolic blood pressure, central obesity, cholesterol and glucose levels. Missing values in the confounders are specified in Table 1. (DOCX 38 kb) [file 12872_2019_1014_MOESM7_ESM.docx]

**Figure S6.** Graphical representation of the results of the association of serum IL8 with the risk of cardiovascular related mortality. Risk estimate are reported in Table 3.

A

B

C
